# Paper-based gamification to enhance competency-based education in an obstetrics clerkship

**DOI:** 10.1186/s12909-026-09725-3

**Published:** 2026-06-15

**Authors:** Yasushi Umezaki, Ai Shinto, Natsumi Yamashita, Mizuki Kobayashi, Mika Fujii, Ayumi Tsuruhisa, Kaoru Okugawa

**Affiliations:** Department of Obstetrics and Gynecology, Faculty of Medicine, Saga University, Nabeshima 5-1-1, Saga-City, 849-8501 Saga Japan

**Keywords:** Competency-based medical education, Clinical clerkship, Gamification, Obstetrics and gynecology, Self-determination theory

## Abstract

**Background:**

Clinical clerkships are essential for competency-based medical education (CBME). However, students’ learning opportunities often vary by case availability, rotation timing, and supervisory practices. Gamification may support learner engagement, but many interventions rely on digital platforms that can be difficult to implement in busy clinical environments. This study evaluated whether a low-resource, paper-based gamification tool could support structured learning in an undergraduate obstetrics and gynecology clerkship.

**Methods:**

This quasi-experimental study included 102 fifth-year medical students who completed a required obstetrics and gynecology clerkship at Saga University between October 2023 and September 2024. Students were assigned by the institutional rotation schedule to either a conventional clerkship group (*n* = 50) or an intervention group using a paper-based “stamp rally” (*n* = 52). The intervention consisted of 20 observable clinical tasks mapped to clerkship competencies and requiring instructor verification. Outcomes included self-reported satisfaction, sense of achievement, self-efficacy, self-directed learning, task completion rate, written examination score, and free-text comments.

**Results:**

Compared with the conventional group, the intervention group reported higher satisfaction (4.3 ± 0.6 vs. 3.6 ± 0.7, *p* < 0.01), sense of achievement (4.4 ± 0.5 vs. 3.5 ± 0.6, *p* < 0.01), self-efficacy (4.2 ± 0.6 vs. 3.7 ± 0.7, *p* < 0.05), and self-directed learning (4.3 ± 0.5 vs. 3.6 ± 0.7, *p* < 0.01). The intervention group also had a higher documented task completion rate (92.3% ± 7.3% vs. 75.9% ± 10.5%, *p* < 0.01) and higher short-term written examination scores (85.0 ± 5.9 vs. 78.2 ± 6.8, *p* < 0.05). Free-text comments suggested that the intervention clarified expectations, promoted proactive engagement, and provided visible progress feedback.

**Conclusions:**

A paper-based gamification tool was associated with higher motivation-related outcomes, greater documented completion of listed clinical tasks, and higher short-term written examination scores in an undergraduate obstetrics and gynecology clerkship. By translating clerkship objectives into visible and verifiable activities, this low-resource approach may help operationalize CBME-oriented clinical learning. Further multicenter and longitudinal studies are needed to evaluate whether improvements in engagement and task completion translate into durable competency development and workplace-based performance.

**Supplementary Information:**

The online version contains supplementary material available at 10.1186/s12909-026-09725-3.

## Background

Clinical clerkships are a core component of undergraduate medical education because they provide students with opportunities to develop clinical reasoning, procedural skills, communication, professionalism, and participation in patient care [1]. In obstetrics and gynecology clerkships, learning often occurs through direct exposure to outpatient care, labor and delivery, inpatient management, surgical procedures, and interprofessional teamwork. However, the educational value of clerkships is strongly influenced by daily clinical circumstances, including patient volume, case mix, timing of rotations, supervisory practices, and the degree to which students are invited or able to participate. Consequently, students may complete the same clerkship with substantially different learning experiences.

Competency-based medical education (CBME) aims to define expected outcomes, clarify progression, and align educational activities with observable competencies [2, 3]. Entrustable professional activities and workplace-based assessments further emphasize the importance of authentic clinical tasks and direct observation in the workplace [4, 5]. Nevertheless, CBME frameworks alone do not fully solve a persistent problem in clinical clerkships: the inconsistency of opportunities to engage in the clinical activities required for competency development. In other words, although CBME can clarify what students are expected to achieve, practical tools are required to help students and instructors operationalize these expectations within busy and unpredictable clinical environments.

This challenge is particularly relevant in obstetrics and gynecology education. Clinical exposure in this field depends heavily on unpredictable events, such as deliveries, emergency presentations, operative schedules, and patient consent for student participation. These contextual factors can create inequity in learning opportunities and uncertainty regarding what students should actively seek during the rotation. Therefore, simple, transparent, and feasible strategies are needed to structure clinical learning without adding substantial burden to students, instructors, or clinical services.

Gamification, defined as the use of game design elements in non-game contexts, has been increasingly applied in medical and health professions education [6]. Elements such as goal setting, progress visualization, immediate feedback, and task completion can enhance engagement and motivation. Systematic reviews suggest that gamification may improve learning behaviors and, in some settings, learning outcomes in health professions education [7–9]. Recent work has also explored gamification in medical students’ diagnostic decision-making and awareness of medical costs [10]. However, many gamification interventions in medical education have relied on digital platforms, online modules, simulation-based systems, or dedicated software. Although these approaches may be effective, they may also require financial resources, technical infrastructure, faculty training, and administrative support that are not always available in authentic clinical settings.

Paper-based gamification may offer a pragmatic alternative or complement to digital approaches. A paper-based tool can make learning objectives visible, convert broad competencies into concrete clinical tasks, and allow students to track their own progress during the clerkship. It can also create brief but meaningful interactions between students and instructors when task completion is verified. Such an approach may be especially useful in resource-limited or high-workload clinical environments because it does not require electronic infrastructure and can be adapted easily to local curricula.

Self-Determination Theory (SDT) provides a useful framework for understanding how such an intervention may influence learner engagement. According to SDT, autonomous motivation is supported when learning environments enhance autonomy, competence, and relatedness [11]. In medical education, SDT has been used to explain how learning environments, teacher support, and educational design can influence students’ motivation and engagement [12, 13]. In the present intervention, autonomy was supported by allowing students to identify and pursue tasks during the clerkship; competence was supported through visible progress tracking and completion of achievable clinical activities; and relatedness was supported through instructor verification and interaction with clinical teams. Thus, the paper-based “stamp rally” was designed not merely as a reward system, but as a low-resource educational structure intended to support motivation, clarify expectations, and promote active participation in clinical learning.

Despite growing interest in gamification in medical education, evidence remains limited regarding non-digital, paper-based gamification embedded in authentic clinical clerkships. In particular, few studies have examined whether such an approach can help operationalize CBME principles in obstetrics and gynecology education. Although recent reviews have addressed gamification in health professions education and midwifery education, the role of low-resource paper-based tools in undergraduate clinical clerkships remains underexplored [7, 8, 14]. Therefore, this study evaluated a paper-based gamification program, the “stamp rally,” implemented during an undergraduate obstetrics and gynecology clerkship. The authors hypothesized that this intervention would be associated with higher motivation-related outcomes, greater documented completion of core clinical tasks, and higher short-term written examination performance by making learning expectations explicit and supporting autonomy, competence, and relatedness in the clinical learning environment.

## Methods

### Study design and theoretical framework

This quasi-experimental study evaluated a paper-based gamification program implemented during a required undergraduate obstetrics and gynecology clerkship. The study compared students who completed the conventional clerkship with those who completed the clerkship after the introduction of the paper-based “stamp rally” intervention.

The intervention was designed using SDT as a conceptual framework [11–13]. Specifically, the stamp rally was intended to support autonomy by allowing students to identify and pursue clinical tasks during the rotation, competence by making task progression visible, and relatedness by requiring brief instructor verification and interaction with clinical teams. The intervention also reflected CBME principles by translating broad clerkship objectives into observable task-based activities [2–5].

Given that allocation was determined by the institutional rotation schedule, randomization was not feasible. This design introduced the risk of systematic bias: students who completed the clerkship before implementation of the stamp rally formed the conventional group, whereas those who completed it after implementation formed the intervention group. Differences in rotation timing, seasonal variation in patient volume, case mix, and changes in instructor experience over the academic year may therefore have influenced the results independently of the intervention. These potential confounders are discussed in the Limitations section. Therefore, the study was designed as a pragmatic educational evaluation conducted in an authentic clinical learning environment, and its findings should be interpreted accordingly.

### Setting and participants

The study was conducted at the Department of Obstetrics and Gynecology, Saga University Hospital, Japan, between October 2023 and September 2024. Saga University School of Medicine offers a six-year undergraduate medical program with an annual intake of approximately 100 students and a total enrollment of approximately 600 students across all year levels. Clinical clerkships are conducted during the fifth and sixth years of the curriculum. The obstetrics and gynecology clerkship is a required two-week rotation completed by all fifth-year medical students as part of their clinical training.

All fifth-year medical students who completed the obstetrics and gynecology clerkship during the study period were eligible for inclusion. Students were assigned to rotation groups according to the predetermined institutional schedule. Students who completed the clerkship before implementation of the stamp rally were included in the conventional group, whereas those who completed the clerkship after implementation were included in the intervention group.

### Educational context and conventional clerkship

The conventional obstetrics and gynecology clerkship consisted of clinical exposure to outpatient care, inpatient management, labor and delivery room activities, perioperative care, surgical observation or assistance, case discussions, and clinical conferences. Students were expected to participate in clinical activities under faculty and resident supervision.

In the conventional clerkship, learning objectives were provided as part of the standard curriculum. However, students’ actual opportunities to experience specific clinical activities depended on patient volume, case mix, timing of the rotation, operating room schedules, deliveries, patient consent, and supervisor availability. Students received routine verbal guidance from instructors, but no structured task completion sheet or progress visualization tool was used.

### Intervention: paper-based stamp rally

Students in the intervention group received a paper-based stamp rally sheet at the beginning of the clerkship. The sheet contained 20 predefined clinical learning tasks mapped to core clerkship competencies. Each task represented an observable activity that could be completed during routine obstetrics and gynecology training.

Students were instructed to actively seek opportunities to complete the listed tasks during the clerkship. After completing each task, students asked a supervising faculty member, resident, midwife, nurse, or other designated clinical instructor to verify the activity. Verification was recorded by placing a stamp or signature on the sheet.

The stamp rally was not used as a high-stakes assessment and did not directly determine students’ clerkship grades. Rather, it was implemented as a formative educational tool to clarify expectations, encourage active participation, support progress monitoring, and promote brief interactions with instructors.

### Development of the 20 clinical tasks

The 20 clinical tasks were developed by faculty members in the Department of Obstetrics and Gynecology based on institutional clerkship objectives, expected undergraduate competencies, and common clinical learning opportunities available in the department. The task list was designed to cover major domains of obstetrics and gynecology clerkship training, including obstetric assessment, gynecologic examination, labor and delivery care, perioperative participation, interprofessional communication, clinical reasoning, feedback, and reflective learning.

The tasks were selected through discussion among faculty members who regularly supervised medical students during the clerkship. Selection criteria included educational relevance, feasibility within the clerkship period, observability in routine clinical settings, and suitability for formative instructor verification. The final list was approved by the clerkship leadership team before implementation. The complete list of the 20 tasks, corresponding domains, educational objectives, and verification methods is provided in Additional file 1 to enhance reproducibility.

### Instructor verification procedures

Instructor verification was used to confirm that students had participated in or completed each task. Before implementation, faculty members, residents, and other clinical instructors involved in student supervision were informed of the purpose of the stamp rally and the expected verification process. Instructors were asked to verify task completion only when they had directly observed the activity or had sufficient knowledge that the student had participated appropriately.

Formal rater training or calibration session was not conducted. Therefore, variability in instructor thresholds for verification may have occurred. To minimize inconsistency, the tasks were designed as simple observable activities rather than complex summative competency judgments. Instructor verification was intended to function as formative confirmation and feedback rather than as a high-stakes assessment of entrustment.

### Outcome measures

The primary educational outcomes were task completion rate and post-clerkship written examination score. Secondary outcomes included students’ self-reported satisfaction, sense of achievement, self-efficacy, and self-directed learning.

Self-reported outcomes were assessed using a post-clerkship questionnaire developed for this study. Each item was rated on a 5-point Likert-scale, with higher scores indicating more positive responses. The questionnaire was designed to evaluate motivation-related outcomes relevant to the intervention. Given that the questionnaire was developed locally and was not a previously validated instrument, the results were interpreted as exploratory self-reported outcomes [15, 16]. The questionnaire items are provided in Additional file 2.

Task completion rate was calculated as the proportion of the listed clinical task categories completed by each student. In the intervention group, task completion was assessed prospectively using the stamp rally sheet, on which instructors placed stamps or signatures to verify each activity. In the conventional group, completion of activities corresponding to the 20 task categories was retrospectively assessed using the standard clerkship activity record maintained by each student. This difference in documentation method represented an important limitation of the comparison: the prospective and structured nature of stamp rally recording in the intervention group may have systematically increased the accuracy and completeness of activity documentation relative to the retrospective activity record used in the conventional group. Therefore, the observed difference in task completion rate was interpreted with particular caution, as it may partly reflect differences in documentation systems rather than differences in actual clinical exposure or participation. Written examination performance was evaluated using the end-of-clerkship written examination. Scores were reported on a 100-point scale.

### Written examination development

The written examination consisted of 25 items developed by faculty members in the Department of Obstetrics and Gynecology using a content blueprint aligned with the clerkship curriculum. The blueprint covered major domains of undergraduate obstetrics and gynecology education, including normal pregnancy and antenatal care, obstetric complications, labor and delivery care, benign gynecology, gynecologic oncology, reproductive endocrinology and infertility, and emergency and perioperative care.

Items were reviewed by multiple faculty members for content relevance, clarity, cognitive level, and alignment with learning objectives. The examination was used as a short-term measure of knowledge acquisition at the end of the clerkship. Formal psychometric validation was not performed. Therefore, the written examination results should be interpreted as short-term academic outcomes rather than comprehensive evidence of clinical competence [17].

Item-level data were not readily available for formal reliability testing; therefore, Cronbach’s alpha could not be calculated for this revision. This limitation was considered when interpreting the written examination results. The written examination blueprint is provided in Additional file 2.

### Qualitative data analysis

Free-text responses from the post-clerkship questionnaire were analyzed using inductive content analysis [18]. Two investigators independently reviewed all responses and generated initial codes. Similar codes were grouped into broader categories through iterative discussion. Disagreements were resolved through consensus. The final themes were reviewed by the research team to ensure that they reflected the students’ comments and the educational aims of the intervention.

Given that the qualitative component was based on brief written comments rather than in-depth interviews or focus groups, formal assessment of theoretical saturation was not performed. The qualitative analysis was therefore used to supplement and contextualize the quantitative findings rather than to generate a comprehensive theory of learner experience. The qualitative coding framework is provided in Additional file 3.

### Ethical considerations

This study was approved by the Ethics Committee of Saga University (Approval No. R7-46). The study was conducted in accordance with the principles of the Declaration of Helsinki and the Ethical Guidelines for Medical and Health Research Involving Human Subjects in Japan. An opt-out consent procedure was used in accordance with institutional policy. The requirement for written informed consent was waived by the Ethics Committee because the study involved analysis of anonymized educational data and posed minimal risk to participants. All data were anonymized before analysis, and participation in the study did not influence students’ clerkship grades or academic evaluations.

### Statistical analysis

Statistical analyses were conducted using JMP Student Edition 18.2.1 (SAS Institute, Cary, NC, USA). Continuous variables were summarized as means and standard deviations. Categorical variables were summarized as frequencies and percentages.

Between-group comparisons were performed using independent-samples t-tests for continuous variables and chi-square tests or Fisher’s exact tests for categorical variables, as appropriate. Effect sizes were calculated using Cohen’s *d* for continuous outcomes. Statistical significance was set at 5% alpha error level (*p* < 0.05).

Since this was a pragmatic quasi-experimental study based on a predetermined rotation schedule, no a priori sample size calculation was performed. Adjustment for potential confounders such as rotation period, instructor variability, and patient volume was not performed because detailed individual-level data on these factors were not available. Therefore, the findings were interpreted with caution, and residual confounding was considered in the Discussion section.

No formal correction for multiple comparisons was applied because the subjective outcomes were prespecified and conceptually related exploratory measures. However, this analytical decision was acknowledged as a limitation, and interpretation focused on the overall pattern of findings rather than isolated *p*-values.

## Results

### Participant characteristics

A total of 102 fifth-year medical students completed the required obstetrics and gynecology clerkship during the study period and were included in the analysis. Of these, 50 students completed the conventional clerkship and 52 students completed the clerkship with the paper-based stamp rally intervention. No eligible students were excluded from the analysis. Baseline characteristics are shown in Table 1. Sex distribution and pre-clerkship written examination scores were comparable between the conventional and intervention groups. No statistically significant differences were observed in the measured baseline variables (Table 1).

**Table 1:** Participants’ baseline characteristics

Characteristic	Conventional group (*n* = 50)	Intervention group (*n* = 52)	*p*-value
Female, *n* (%)	23 (46.0)	24 (46.2)	0.98
Male, *n* (%)	27 (54.0)	28 (53.8)	0.98
Pre-clerkship written examination score	52.9 ± 18.6	54.1 ± 19.4	0.75

### Self-reported outcomes

Students in the intervention group reported higher scores across all self-reported outcomes than those in the conventional group (Table 2). Mean satisfaction was higher in the intervention group than in the conventional group (4.3 ± 0.6 vs. 3.6 ± 0.7, *p* < 0.01, Cohen’s *d* = 1.07). Sense of achievement was also higher in the intervention group (4.4 ± 0.5 vs. 3.5 ± 0.6, *p* < 0.01, Cohen’s *d* = 1.55).

**Fig. 1:**
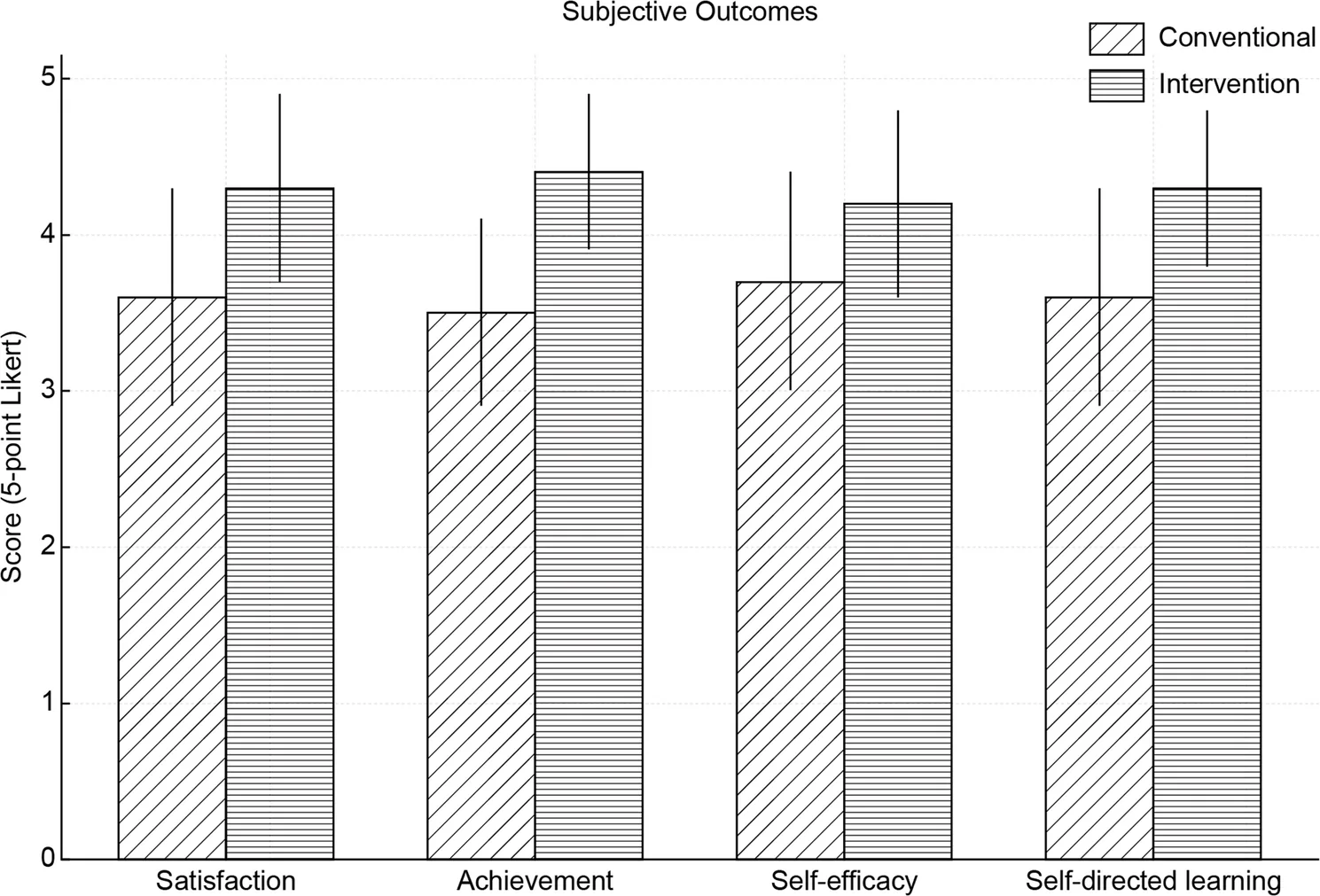
Self-reported outcomes. Mean scores for self-reported satisfaction, sense of achievement, self-efficacy, and self-directed learning. Scores were based on 5-point Likert-scale items. The intervention group reported higher scores across all four domains

Self-efficacy was higher among students in the intervention group than among those in the conventional group (4.2 ± 0.6 vs. 3.7 ± 0.7, *p* < 0.05, Cohen’s *d* = 0.75). Self-directed learning was also higher in the intervention group (4.3 ± 0.5 vs. 3.6 ± 0.7, *p* < 0.01, Cohen’s *d* = 1.15). These results are summarized in Fig. 1.

**Table 2:** Comparison of self-reported outcomes

Outcome	Conventional group (*n* = 50)	Intervention group (*n* = 52)	*p*-value	Cohen’s d
Satisfaction	3.6 ± 0.7	4.3 ± 0.6	< 0.01	1.07
Sense of achievement	3.5 ± 0.6	4.4 ± 0.5	< 0.01	1.55
Self-efficacy	3.7 ± 0.7	4.2 ± 0.6	< 0.05	0.75
Self-directed learning	3.6 ± 0.7	4.3 ± 0.5	< 0.01	1.15

Values are presented as mean ± standard deviation. All outcomes were assessed using 5-point

Likert-scale items, with higher scores indicating more positive responses

### Task completion and written examination performance

**Fig. 2:**
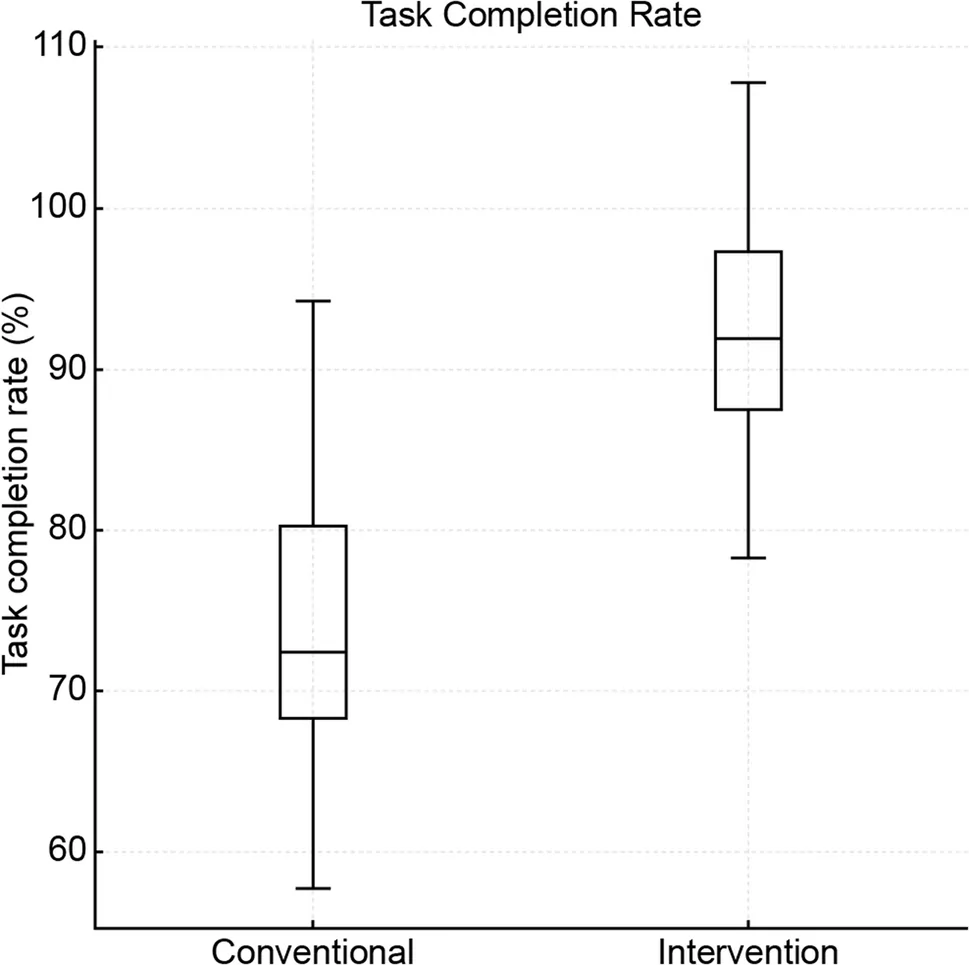
Task completion rates. Distribution of task completion rates in the conventional and intervention groups. The intervention group completed a higher proportion of listed clinical tasks

**Fig. 3:**
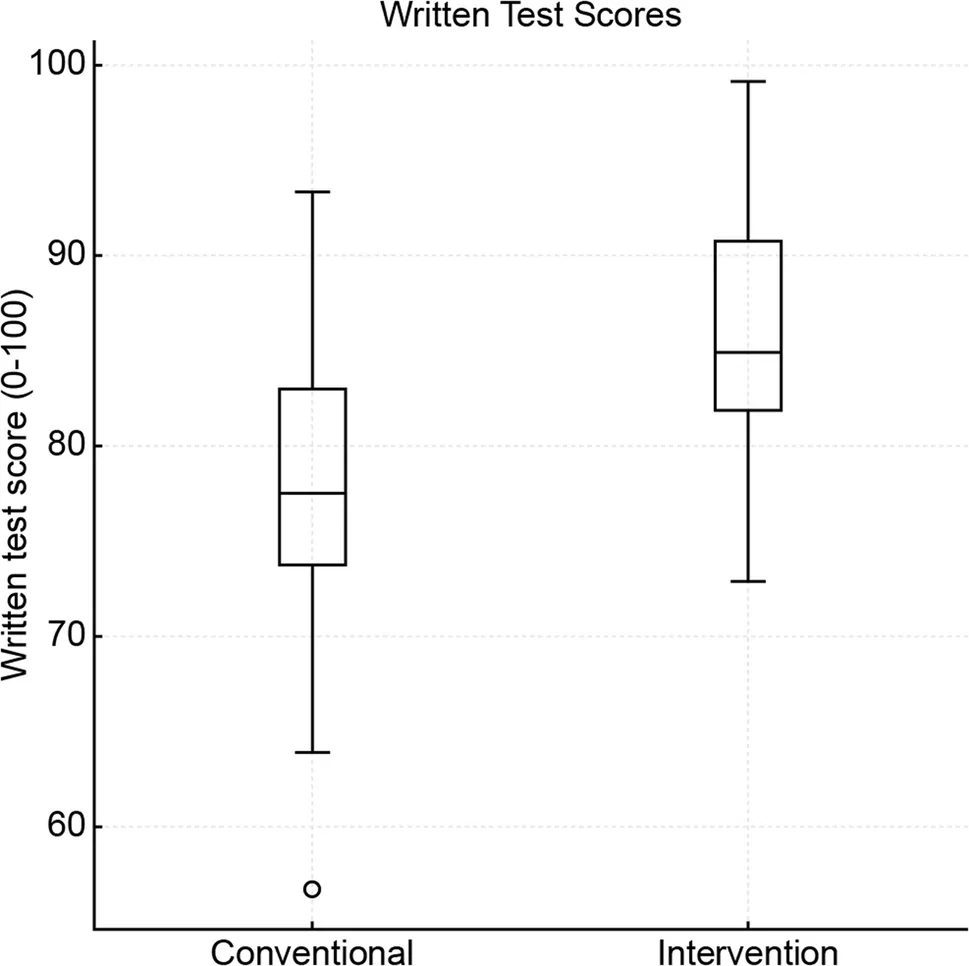
Written examination scores. Comparison of post-clerkship written examination scores between groups. Scores are shown on a 100-point scale

Students in the intervention group had a higher documented completion rate for listed clinical tasks than students in the conventional group (92.3% ± 7.3% vs. 75.9% ± 10.5%, *p* < 0.01, Cohen’s *d* = 1.73) (Table 3). The distribution of task completion rates is shown in Fig. 2. Post-clerkship written examination scores were also higher in the intervention group than in the conventional group (85.0 ± 5.9 vs. 78.2 ± 6.8 on a 100-point scale, *p* < 0.05, Cohen’s *d* = 1.07). The comparison of written examination scores is shown in Fig. 3.

**Table 3:** Comparison of task completion and written examination performance

Outcome	Conventional group (*n* = 50)	Intervention group (*n* = 52)	*p*-value	Cohen’s d
Task completion rate, %	75.9 ± 10.5	92.3 ± 7.3	< 0.01	1.73
Written examination score	78.2 ± 6.8	85.0 ± 5.9	< 0.05	1.07

### Qualitative findings

Free-text responses from the intervention group provided additional context regarding students’ experiences with the stamp rally. Inductive content analysis identified three major themes: clarity of learning expectations, proactive engagement, and motivation through progress visualization. Open-ended responses from students in the conventional group were also reviewed and are presented in Table 4 as contextual contrast; formal coding was applied to intervention group responses only. Conventional group responses frequently described uncertainty regarding expected activities and perceived inconsistency in access to core learning opportunities, providing contextual support for the intervention’s rationale. Representative intervention group comments and a contextual summary from the conventional group are shown in Table 4.

**Table 4:** Themes identified from free-text responses

Theme	Description	Representative comment or contextual summary
Clarity of learning expectations	The task list helped students understand expected learning activities during the clerkship.	The stamp rally helped me understand what I should experience during the clerkship.
Proactive engagement	Students described becoming more willing to seek opportunities for observation or participation.	It made it easier for me to ask whether I could observe or participate in clinical activities.
Motivation through progress visualization	Accumulating stamps provided visible feedback and a sense of progress.	Seeing the number of stamps increase encouraged me and gave me a sense of achievement.
*Uncertainty in conventional clerkship (contextual contrast)*	Students in the conventional group described uncertainty regarding expected activities and perceived inconsistency in access to core learning opportunities.	“I was sometimes unsure what I should do during the clerkship, and learning opportunities varied.”

Formal inductive coding was applied to intervention group responses only. Conventional group responses were reviewed to provide contextual contrast and are presented descriptively. The contextual summary from the conventional group is a composite paraphrase reflecting the predominant content of those responses

### Exploratory subgroup and rotation-related analyses

Sex-based subgroup analyses showed no significant differences in subjective or objective outcomes within each group. The direction of the intervention effect was consistent across sex subgroups. Exploratory review of outcomes by rotation order did not identify a clear clustering pattern. However, because the study was not randomized and detailed data on patient volume, instructor assignment, and case exposure were not available, the possibility of rotation-related confounding could not be excluded.

## Discussion

### Principal findings

This study evaluated a low-resource, paper-based gamification program implemented during an undergraduate obstetrics and gynecology clerkship. The results indicated that students in the intervention group reported higher motivation-related outcomes, had a greater documented completion rate for listed clinical tasks, and achieved higher short-term written examination scores than students in the conventional group. Qualitative findings suggested that the structured task list clarified learning expectations, encouraged students to seek clinical opportunities more actively, and provided a visible sense of progress during the clerkship.

The main contribution of this study is not that a paper-based stamp system alone improved all dimensions of clinical competence, but that a simple and feasible educational tool may help operationalize competency-based learning in a clinical environment where opportunities are often variable and difficult to standardize. The findings suggest that making expected activities explicit and visible may support student engagement during clinical clerkships. Within Kirkpatrick’s four-level evaluation framework [19], the outcomes assessed here corresponded primarily to the first two levels: learner reaction (self-reported motivation and satisfaction) and learning (short-term written examination performance and task participation). Higher-level outcomes, including behavioral change in subsequent clinical practice and impact on patient care, require evaluation in future longitudinal studies.

### Interpretation through self-determination theory

The findings can be interpreted through the lens of SDT. The stamp rally was designed to support autonomy, competence, and relatedness, and the observed outcomes were broadly consistent with each of these components. Autonomy may have been supported by allowing students to identify remaining tasks and choose when to seek opportunities during the rotation; this was consistent with higher self-directed learning scores in the intervention group (Cohen’s *d* = 1.15). Competence may have been supported by visible progress tracking and repeated completion of achievable clinical activities; this corresponded with the higher sense of achievement reported by intervention students (Cohen’s *d* = 1.55). Relatedness may have been supported by the requirement for instructor verification, which created brief interactions between students and clinical supervisors; this may have partially explained the higher satisfaction scores observed (Cohen’s *d* = 1.07).

The qualitative findings were consistent with this interpretation and can be mapped directly onto the three SDT components. The theme of clarity of learning expectations corresponds to the CBME-oriented function of the stamp rally: by translating broad clerkship objectives into observable tasks, the tool reduced uncertainty about what students were expected to achieve, a prerequisite for autonomous engagement. The theme of proactive engagement corresponds to the autonomy component of SDT: students reported actively seeking clinical opportunities because the task list offered a concrete basis for initiating participation with supervisors. The theme of motivation through progress visualization corresponds to the competence component: accumulating stamps provided visible evidence of progress and a sense of achievement that sustained engagement throughout the rotation. Additionally, the act of seeking instructor verification to receive a stamp created brief but structured interactions with clinical supervisors, which may have supported the relatedness component of SDT and contributed to the higher satisfaction scores observed in the intervention group.

However, this interpretation should be considered with caution. The study did not directly measure SDT constructs using validated instruments such as the Basic Psychological Needs Satisfaction Scale or the Situational Motivation Scale. Therefore, SDT should be regarded as an explanatory framework for interpreting the observed findings rather than as a directly tested mediation model. The alignment between intervention components and SDT constructs is theoretical, and alternative explanations, including increased instructor attention, novelty effects, or Hawthorne effects, cannot be excluded. The task-based structure also reflects Kolb’s experiential learning cycle, in that students engaged in concrete clinical experiences, observed or participated in procedures, reflected through the act of seeking verification, and connected these activities to explicit learning objectives [20].

### Relationship to competency-based medical education

The stamp rally also reflected several principles of CBME. CBME emphasizes clearly defined outcomes, observable activities, formative feedback, and progression toward clinical competence. In this study, broad clerkship objectives were translated into 20 observable clinical tasks that students could pursue during routine clinical training.

This task-based structure may have reduced uncertainty about what students were expected to experience. In particular, obstetrics and gynecology clerkships are influenced by unpredictable clinical events such as deliveries, emergency cases, surgical schedules, and patient consent for student involvement. In such settings, students may benefit from explicit guidance regarding core activities that should be sought during the rotation.

Nevertheless, this study did not directly measure whether variability in clinical exposure was reduced. The higher task completion rate indicates that students in the intervention group completed more listed activities, but it does not prove that the overall distribution of clinical experiences became more equitable. Future studies should directly assess variation in case exposure, number of observed or performed procedures, quality of supervision, and opportunities for participation.

### Interpretation of task completion

Task completion was one of the most prominent differences between groups, yielding the largest observed effect size in this study (Cohen’s *d* = 1.73). However, this finding requires careful interpretation for two reasons. First, a completed stamp may indicate exposure, participation, or verification of an activity, but it does not necessarily demonstrate independent performance, entrustment, or durable skill acquisition [17, 21]. Second, task completion was assessed using different methods in the two groups: prospective stamp rally documentation in the intervention group and retrospective activity record review in the conventional group. This methodological asymmetry may have contributed to the observed difference in task completion rate, independent of any true difference in clinical participation. Therefore, the effect size for task completion should be interpreted with this limitation in mind.

The increase in task completion may also partly reflect the design of the intervention itself. Since the stamp rally made tasks visible and required verification, students may have been more likely to pursue and document activities. This may represent genuine improvement in structure and engagement, but it may also reflect compliance with an explicit checklist. Therefore, task completion should be interpreted as an intermediate educational outcome reflecting participation and structured engagement, rather than as proof of deeper learning or clinical competence.

This distinction is important for positioning the intervention appropriately. The stamp rally may serve as a formative tool that promotes participation and learning awareness. However, it should not replace workplace-based assessment, direct observation of performance, or entrustment decisions [21].

### Written examination performance and academic outcomes

Students in the intervention group achieved higher post-clerkship written examination scores. This result suggests that the intervention may have supported short-term knowledge acquisition, possibly by increasing students’ engagement with clinical tasks and helping them connect practical experiences with curricular objectives.

However, this outcome should be interpreted specifically as short-term written examination performance. The examination did not assess long-term retention, clinical reasoning in authentic practice, procedural competence, or progression toward entrustment. Therefore, the findings should not be overgeneralized as evidence of improved clinical performance or comprehensive academic achievement [17].

The effect sizes observed across all outcomes in this study were large by conventional standards, with Cohen’s *d* ranging from 0.75 to 1.73. Several mechanisms may have contributed to overestimation and readers should interpret these values with appropriate caution. The quasi-experimental design and unmeasured confounding may have inflated between-group differences. Self-reported outcomes may additionally have been influenced by social desirability bias, novelty effects, and the Hawthorne effect. The task completion rate may also have been inflated by the asymmetric documentation methods described above. For the written examination, ceiling effects, differences in case exposure during specific rotation periods, and increased instructor attention in the intervention group are further potential sources of bias. The true effect sizes in future randomized or multicenter studies with more rigorous controls may therefore be smaller than those reported here. Future studies should include longitudinal assessments, workplace-based performance measures, and validated motivational instruments.

### Comparison with digital gamification

Previous gamification studies in medical education have often used digital platforms, online modules, mobile applications, or simulation-based systems. These approaches can provide automated feedback, data tracking, and interactive learning experiences. However, digital systems may require technical infrastructure, financial resources, faculty training, and administrative support. In busy clinical environments, digital tools may also be difficult to integrate into routine bedside teaching.

This study suggests that paper-based gamification may offer a pragmatic alternative or complement to digital approaches. A paper-based tool can be implemented with minimal cost, requires no electronic infrastructure, and can be adapted to local clerkship objectives. It may also encourage face-to-face interaction because task completion requires instructor verification.

However, this study did not directly compare paper-based and digital gamification. Therefore, conclusions regarding superiority or equivalence should be avoided. Paper-based and digital approaches may have different strengths. Digital systems may be better suited for longitudinal tracking and data aggregation, whereas paper-based tools may be easier to implement rapidly in resource-limited or high-workload clinical settings. Future comparative studies are needed to clarify which approach is most appropriate for different educational contexts.

### Practical implications and implementation feasibility

The intervention has several practical implications. First, converting clerkship objectives into observable tasks may help students understand what they should actively seek during clinical rotations. Second, visible progress tracking may support motivation and self-regulated learning. Third, instructor verification may create brief opportunities for feedback and interaction.

From an implementation perspective, the paper-based format required minimal financial cost and no digital infrastructure. This may be advantageous for institutions with limited technical resources or for departments where faculty workload makes complex educational systems difficult to maintain. The intervention can also be adapted to different specialties by modifying the task list according to local competencies and available clinical experiences.

Meanwhile, implementation requires attention to faculty workload and consistency. Even brief stamp verification may become burdensome if expectations are unclear or if many students request verification simultaneously. Faculty and residents should understand that the stamp rally is a formative learning tool rather than a high-stakes assessment. Clear task definitions, simple verification criteria, and orientation for instructors may improve feasibility and reduce variability in implementation.

### Limitations

This study has several limitations. First, the quasi-experimental design relied on a predetermined rotation schedule rather than random allocation, with the conventional group completing the clerkship in the earlier part of the academic year and the intervention group in the later part. This temporal structure introduced several potential confounders. Obstetric case volume and operative exposure may vary seasonally; for example, delivery rates and surgical schedules may differ between autumn and summer rotations. Additionally, supervising residents and faculty members typically accumulate teaching experience over the course of the academic year, which may have improved the quality of supervision available to the intervention group independent of the stamp rally. Students rotating later in the year may also have had greater prior exposure to clinical settings during other clerkships. Although measured baseline characteristics were comparable between groups, these temporal confounders could not be controlled and may have contributed to the observed differences. Age data were not available and therefore could not be included in the baseline comparison.

Second, the study was conducted at a single institution in one obstetrics and gynecology clerkship. Institutional culture, curriculum structure, faculty engagement, patient volume, and student expectations may influence the feasibility and effectiveness of the intervention. Therefore, generalizability to other institutions, specialties, or countries should be examined carefully.

Third, the subjective outcomes were measured using locally developed Likert-scale items rather than previously validated instruments. These outcomes should therefore be interpreted as exploratory self-reported measures. They may also have been influenced by social desirability bias, novelty effects, or the Hawthorne effect.

Fourth, task completion required instructor verification, but formal rater calibration was not performed. Although tasks were designed as simple observable activities rather than high-stakes competency judgments, variability in instructor thresholds for verification may have occurred. More importantly, task completion was assessed using fundamentally different methods in the two groups: prospective stamp rally documentation in the intervention group and retrospective review of standard activity records in the conventional group. This asymmetry is a critical limitation. The prospective and structured format of stamp rally recording is likely to produce more complete and accurate documentation of completed activities than retrospective recall from a general activity log. Consequently, the observed difference in task completion rate (92.3% vs. 75.9%, Cohen’s *d* = 1.73) may overestimate the true difference in clinical participation and should not be interpreted as reflecting solely the educational effect of the intervention itself.

Fifth, the study did not adjust for all potential confounders, including rotation period, instructor variability, patient volume, prior clinical experience, academic performance beyond available baseline measures, or baseline motivation. Moreover, no formal correction for multiple comparisons was applied to the subjective outcomes. These factors may have contributed to overestimation of the intervention effect.

Sixth, the qualitative analysis was based on brief written comments rather than interviews or focus groups. Although two investigators independently coded the responses and resolved discrepancies by consensus, theoretical saturation was not assessed. The qualitative findings should therefore be regarded as contextual support for the quantitative results rather than as a comprehensive exploration of learner experience.

Finally, this study evaluated short-term outcomes only. It did not assess long-term knowledge retention, subsequent workplace performance, procedural skill development, EPA progression, or clinical competence. Future studies should examine whether increased engagement and task completion translate into durable competency development.

### Future directions

Future research should evaluate paper-based gamification in multicenter and multispecialty settings to determine its generalizability. Studies should also include direct measures of clinical exposure, quality of supervision, and workplace-based performance, as well as validated instruments for assessing SDT constructs such as autonomy support and basic psychological need satisfaction. The experiential learning cycle described by Kolb [20] provides an additional theoretical lens for designing future iterations of task-based clerkship tools, emphasizing the importance of structured reflection and conceptualization alongside concrete clinical experience. Randomized or cluster-randomized designs may help reduce allocation bias, although pragmatic designs will remain important for evaluating educational interventions in real clinical settings.

Longitudinal studies are needed to determine whether short-term improvements in motivation, task completion, and written examination scores are associated with later clinical performance or progression toward entrustment. Moreover, comparative studies of paper-based, digital, and hybrid gamification approaches could clarify which format best supports different types of learners, institutions, and clinical environments.

## Conclusions

A low-resource, paper-based gamification tool was associated with higher motivation-related outcomes, greater documented completion of listed clinical tasks, and higher short-term written examination scores in an undergraduate obstetrics and gynecology clerkship. By translating clerkship objectives into visible and verifiable activities, the intervention may help clarify learning expectations and support active engagement in CBME-oriented clinical learning.

These findings should be interpreted cautiously because the study was quasi-experimental, conducted at a single institution, and limited to short-term outcomes. Future multicenter and longitudinal studies are needed to determine whether paper-based gamification contributes to durable competency development and workplace-based performance. 

## Supplementary Information

Additional file 1. Supplementary educational materials. List of 20 stamp rally tasks and corresponding competencies, including clinical domains, educational objectives, and verification methods.

Additional file 2. Supplementary assessment materials. Post-clerkship questionnaire items and written examination blueprint.

Additional file 3. Supplementary qualitative analysis. Qualitative coding framework and representative free-text comments.

## Data Availability

The datasets used and/or analysed during the current study are available from the corresponding author on reasonable request.

## Abbreviations

CBME: Competency-based medical education
EPA: Entrustable professional activity
SDT: Self-Determination Theory
