# Supplementary material for: Paper-based gamification to enhance competency-based education in an obstetrics clerkship
Source: BMC Med Educ. 2026 Jun 15;26:1361. doi: 10.1186/s12909-026-09725-3 (PMC13501658; doi:10.1186/s12909-026-09725-3)
Supplement: Supplementary file 1 [file 12909_2026_9725_MOESM1_ESM.docx]

**Additional file 1: Supplementary educational materials**

Description of data: List of 20 stamp rally tasks and corresponding competencies, including clinical domains, educational objectives, and verification methods.

**Supplementary Table S1.** Stamp rally tasks and corresponding competencies

| **Task No.** | **Original Japanese task** | **English task description** | **Clinical domain** | **Verification method** |
| --- | --- | --- | --- | --- |
| 1 | レオポルド触診法 | Perform or observe Leopold’s maneuvers under supervision | Obstetric assessment | Stamp or signature by instructor |
| 2 | 胎児CTG判読 | Interpret fetal cardiotocography under supervision | Obstetric assessment | Stamp or signature by instructor |
| 3 | 胎児超音波 | Observe or participate in fetal ultrasonography | Obstetric assessment | Stamp or signature by instructor |
| 4 | 妊婦健診見学 | Observe antenatal check-up | Obstetric care | Stamp or signature by instructor |
| 5 | 婦人科内診見学 | Observe gynecologic pelvic examination | Gynecologic examination | Stamp or signature by instructor |
| 6 | 細胞診介助 | Assist with cervical cytology | Gynecologic procedure | Stamp or signature by instructor |
| 7 | 腹腔鏡ドライボックス | Practice laparoscopic skills using a dry box | Surgical simulation | Stamp or signature by instructor |
| 8 | 腹部エコー | Observe or perform abdominal ultrasonography under supervision | Gynecologic/obstetric imaging | Stamp or signature by instructor |
| 9 | 分娩介助 | Assist with delivery care as appropriate | Delivery care | Stamp or signature by instructor |
| 10 | 帝王切開介助 | Assist with cesarean section as appropriate | Obstetric surgery | Stamp or signature by instructor |
| 11 | 手術前手洗い | Perform preoperative surgical scrubbing | Surgical preparation | Stamp or signature by instructor |
| 12 | 皮膚縫合 | Practice or perform skin suturing under supervision | Surgical skill | Stamp or signature by instructor |
| 13 | 尿道カテーテル挿入 | Perform or assist with urinary catheter insertion under supervision | Basic clinical procedure | Stamp or signature by instructor |
| 14 | 多職種カンファ | Attend an interprofessional conference | Interprofessional care | Stamp or signature by instructor |
| 15 | 患者説明の陪席 | Attend patient explanation or counseling | Communication | Stamp or signature by instructor |
| 16 | 医療面接・問診 | Conduct or observe medical interview/history taking | Communication/clinical reasoning | Stamp or signature by instructor |
| 17 | 清潔操作 | Perform sterile technique | Infection control/procedural safety | Stamp or signature by instructor |
| 18 | 症例レポート | Prepare a case report | Clinical reasoning/reflection | Stamp or signature by instructor |
| 19 | 消毒・創傷処置 | Perform or assist with disinfection and wound care | Basic clinical procedure | Stamp or signature by instructor |
| 20 | フィードバック依頼 | Request feedback from an instructor | Feedback/reflection | Stamp or signature by instructor |

Footnote: The stamp or signature indicated formative confirmation of participation or completion, and it was not used as a high-stakes assessment of competence or entrustment.
