# Supplementary material for: Paper-based gamification to enhance competency-based education in an obstetrics clerkship
Source: BMC Med Educ. 2026 Jun 15;26:1361. doi: 10.1186/s12909-026-09725-3 (PMC13501658; doi:10.1186/s12909-026-09725-3)
Supplement: Supplementary file 2 [file 12909_2026_9725_MOESM2_ESM.docx]

**Additional file 2: Supplementary assessment materials**

Description of data: Post-clerkship questionnaire items and written examination blueprint.

**Supplementary Table S2.** Post-clerkship questionnaire items

| **Domain** | **Questionnaire item** | **Response scale** |
| --- | --- | --- |
| Satisfaction | Overall, I was satisfied with the obstetrics and gynecology clerkship. | 1 = strongly disagree to 5 = strongly agree |
| Sense of achievement | I felt a sense of achievement through the clerkship activities. | 1 = strongly disagree to 5 = strongly agree |
| Self-efficacy | I felt more confident in understanding basic obstetrics and gynecology practice after the clerkship. | 1 = strongly disagree to 5 = strongly agree |
| Self-directed learning | I was able to identify and pursue learning opportunities actively during the clerkship. | 1 = strongly disagree to 5 = strongly agree |
| Free-text comment | Please describe what you learned, found useful, or found difficult during the clerkship. | Free text |

The questionnaire was developed locally for this study and was not a previously validated instrument. The items were designed to evaluate motivation-related outcomes relevant to the intervention. Therefore, the questionnaire results were interpreted as exploratory self-reported outcomes.

**Supplementary Table S3.** Blueprint of the end-of-clerkship written examination

| **Domain** | **Main content** | **Cognitive level** | **Number of items** |
| --- | --- | --- | --- |
| Normal pregnancy and antenatal care | Prenatal care, maternal assessment, fetal assessment | Recall/application | 5 |
| Obstetric complications | Hypertensive disorders, preterm labor, fetal growth restriction, obstetric emergencies | Application/reasoning | 2 |
| Labor and delivery care | Labor progress, delivery management, postpartum assessment | Recall/application | 2 |
| Benign gynecology | Menstrual disorders, benign uterine and ovarian diseases, pelvic pain | Recall / application | 2 |
| Gynecologic oncology | Cervical, endometrial, and ovarian cancer; screening and diagnosis | Application/reasoning | 6 |
| Reproductive endocrinology and infertility | Amenorrhea, endocrine disorders, infertility evaluation | Recall/application | 3 |
| Emergency and perioperative care | Acute abdomen, perioperative management, postoperative complications | Application/reasoning | 5 |
| Total |  |  | 25 |

The written examination was developed by faculty members in the Department of Obstetrics and Gynecology using a content blueprint aligned with the clerkship curriculum. Items were reviewed by multiple faculty members for content relevance, clarity, cognitive level, and alignment with learning objectives. Formal psychometric validation was not performed, and item-level data were not readily available for calculating Cronbach’s alpha.
