# Supplementary material for: Paper-based gamification to enhance competency-based education in an obstetrics clerkship
Source: BMC Med Educ. 2026 Jun 15;26:1361. doi: 10.1186/s12909-026-09725-3 (PMC13501658; doi:10.1186/s12909-026-09725-3)
Supplement: Supplementary file 3 [file 12909_2026_9725_MOESM3_ESM.docx]

**Additional file 3: Supplementary qualitative analysis**

Description of data: Qualitative coding framework and representative free-text comments.

**Supplementary Table S4.** Coding framework for free-text responses

| **Theme** | **Initial codes** | **Representative comment** |
| --- | --- | --- |
| Clarity of learning expectations | Clear goals; knowing what to do next; visible task list | The stamp rally helped me understand what I should experience during the clerkship. |
| Proactive engagement | Asking to participate; seeking opportunities; active behavior | It made it easier for me to ask whether I could observe or participate in clinical activities. |
| Motivation through progress visualization | Sense of progress; achievement; motivation to complete tasks | Seeing the number of stamps increase encouraged me and gave me a sense of achievement. |

Formal inductive coding was applied to intervention group responses only. Conventional group responses were reviewed to provide contextual contrast and are presented descriptively in Table 4 of the main manuscript; they were not included in this coding framework. Free-text responses from the intervention group were reviewed independently by two investigators. Initial codes were generated inductively and grouped into themes through discussion. Disagreements were resolved by consensus. Given that the data consisted of brief written comments rather than interviews or focus groups, formal theoretical saturation was not assessed.
